# The Systemic and Cellular Metabolic Phenotype of Infection and Immune Response to *Listeria monocytogenes*

**DOI:** 10.3389/fimmu.2020.614697

**Published:** 2021-02-08

**Authors:** Robert M. Johnson, Adesola C. Olatunde, Lauren N. Woodie, Michael W. Greene, Elizabeth Hiltbold Schwartz

**Affiliations:** ^1^ Department of Biological Sciences, Auburn University, Auburn, AL, United States; ^2^ Department of Nutrition, Dietetics, and Hospitality Management, Auburn University, Auburn, AL, United States

**Keywords:** metabolic phenotype, *Listeria (L.) monocytogenes*, sickness behavior, immunometabolism, life history theory

## Abstract

It is widely accepted that infection and immune response incur significant metabolic demands, yet the respective demands of specific immune responses to live pathogens have not been well delineated. It is also established that upon activation, metabolic pathways undergo shifts at the cellular level. However, most studies exploring these issues at the systemic or cellular level have utilized pathogen associated molecular patterns (PAMPs) that model sepsis, or model antigens at isolated time points. Thus, the dynamics of pathogenesis and immune response to a live infection remain largely undocumented. To better quantitate the metabolic demands induced by infection, we utilized a live pathogenic infection model. Mice infected with *Listeria monocytogenes* were monitored longitudinally over the course of infection through clearance. We measured systemic metabolic phenotype, bacterial load, innate and adaptive immune responses, and cellular metabolic pathways. To further delineate the role of adaptive immunity in the metabolic phenotype, we utilized two doses of bacteria, one that induced both sickness behavior and protective (T cell mediated) immunity, and the other protective immunity alone. We determined that the greatest impact to systemic metabolism occurred during the early immune response, which coincided with the greatest shift in innate cellular metabolism. In contrast, during the time of maximal T cell expansion, systemic metabolism returned to resting state. Taken together, our findings demonstrate that the timing of maximal metabolic demand overlaps with the innate immune response and that when the adaptive response is maximal, the host has returned to relative metabolic homeostasis.

## Introduction

One of the central postulates of life history theory is that certain finite resources must be allocated between growth, reproduction, and maintenance over an animal’s lifespan (1, 2). With limited resources, competitive trade-offs will occur when the demand for one trait is greater than the others (1, 2). Such trade-offs typically occur between immunity, growth, and reproduction (1, 2). Many studies exploring immune-driven trade-offs have relied on non-infectious stimuli that broadly activate the innate (LPS) or adaptive (Keyhole Limpet Hemocyanin, KLH) arms of the immune response (3) (4). Generally, it has been reported that metabolic rate goes up during these immune responses with fever being the most well documented metabolic demand (5–8). Numerous studies have also examined the metabolic changes associated with polymicrobial sepsis using a cecal ligation and puncture model (9). These studies have observed similar outcomes as LPS administration including fever, weight loss, decrease activity, and decreased food and water consumption (9–12).

While many such studies have highlighted systemic trade-offs that occur during model immune responses, we still have much to learn about such trade-offs during live infection. First, the use of pathogen associated molecular patterns (PAMPs), model antigens, or mitogens cannot replicate the progressive stages of live infection (invasion, replication, spread, infection-induced pathology). Secondly, these studies model systemic infections such as sepsis, and thus fail to represent localized infection and inflammation. Finally, many of the metabolic measurements have been performed at isolated timepoints (removing animals from home caging for metabolic measurements, inducing stress), not longitudinally throughout the infection and immune response. To better elucidate the metabolic demands and trade-offs that occur from initial infection through clearance, the systemic metabolism of the host should be monitored longitudinally during live pathogen infection and clearance.

*Listeria monocytogenes* is a Gram positive intracellular foodborne pathogen. Its pathogenicity and the immune response to it have been well documented in a variety of infection routes (13–15). We chose to use the intraperitoneal infection route for this study as it is well documented with regard to kinetics of bacterial burden and immune response and is highly reproducible (16–19). This infection model is characterized by an initially localized infection in the peritoneum that disseminates systemically, but with slower kinetics than the intravenous route (20–23). Using this route of infection, in a dose range from 2x10^3^–2x10^4^, protective T cell responses develop, with little observable sickness at the low end while measurable, but non-life-threatening illness is observed at the high end (24, 25). During the early phase of infection, a robust innate immune response is observed consisting of inflammatory cytokine production and recruitment of neutrophils and monocytes to the liver and spleen (13, 25–31). Bacterial burden in the spleen and liver also peaks during this phase (21, 28, 29). On the heels of the innate response, the adaptive immune response (predominantly T cells) undergoes rapid expansion (13, 22, 23, 32). The *Listeria-*specific T cell response peaks around day 7–9 then undergoes contraction, establishing a memory population by day 14 (22, 23, 32, 33). While *L. monocytogenes* infection has been well characterized, the metabolic status of cells responding to this infection remains to be determined.

Under resting conditions, cells primarily use oxidative phosphorylation (OXPHOS) for energy when oxygen is available. Under anaerobic conditions, cells utilize fermentation for energy. However, in 1924 Dr. Otto Warburg observed unique metabolic patterns in cancer cells. He determined that cancerous cells use lactic acid fermentation for energy production in the presence or absence of oxygen (34, 35), a phenomenon termed the “Warburg” effect (36). In recent years, there has been a surge in the study of cellular metabolic status of immune cells, a field now known as immunometabolism (37, 38). Several studies have now established that cells of the immune system differ in metabolic processes based on their activation state (37, 38).

Quiescent cells of the innate immune system primarily use OXPHOS for energy; however, upon Toll Like Receptor activation, these cells shift to glycolysis (39–41). Naïve T cells utilize fatty acid oxidation and OXPHOS for energy (42, 43). Upon activation, if the cell becomes an effector T cell, it utilizes OXPHOS for energy, while utilizing glycolysis and glutaminolysis for cellular proliferation and cytokine production (44–47). However, if the cell becomes a memory cell, it will primarily use fatty acid oxidation (48, 49). While these changes in cellular metabolism of the immune system have now been well documented, most of these studies used either PAMPs or non-specific T cell activation, thus, the immunometabolic changes during live infection remain poorly understood.

Our present study aims to provide an integrated examination of systemic and cellular metabolism over the time course of a pathogenic infection with and primary immune response to *L. monocytogenes.* We determined that significant changes in systemic metabolism occurred only above a threshold level of infection, and these changes occurred simultaneously with the innate immune response. We also observed an increase in a glucose transporter primarily in cells expressing Ly6C or Ly6G during this time. Additionally, in our infection model, during the period of maximal T cell expansion, this adaptive immune response did not cause detectable changes in host behavior or systemic metabolism.

## Materials and Methods

### Mice

C57BL/6J mice were obtained from The Jackson Laboratory. The mice used in these studies were female, between 8 and 12 weeks old and were age-matched for each experiment. We chose to initiate this project in female mice so that they may be readily extended into future studies on reproductive success. All mice were maintained in a specific pathogen-free (SPF) facility and in full compliance with the Institutional Care and Use Committee of Auburn University regarding the use of animals.

### *Listeria monocytogenes* Infection and Bacterial Enumeration

Wild type *Listeria monocytogenes* (Lm-10403s, obtained from Dr. Daniel Portnoy, University of California, Berkeley) was grown in brain-heart infusion (BHI) broth overnight at 37°C to an OD_600_ of 1.0. The overnight culture (1 ml) was centrifuged, resuspended in PBS, and washed twice in PBS. Mice were injected intraperitoneally with either 2.5x10^4^ CFU/ml (High Dose) or 1x10^4^ CFU/ml (Low Dose) or an equal volume of PBS diluent (uninfected control). The infectious dose was confirmed by plating dilutions of the inoculum on BHI agar, and colonies were counted after incubation at 37°C for 18–24 h. Spleens and livers were homogenized and lysed in 3 ml sterile dH_2_O, serial dilutions of the homogenates were plated on BHI agar, and colonies were counted after incubation at 37°C for 18–24 h.

### Metabolic Phenotyping, Food, and Water Intake

To assess metabolic phenotype, Promethion metabolic cages (Sable Systems, Las Vegas, NV) were used as previously described (50, 51). Briefly, animals were individually housed in the metabolic cages throughout the 12-day experiment. Activity was measured by Promethion XYZ Beambreak Activity Monitors and was determined by consecutive adjacent beam breaks in the X, Y, and Z planes. Quiet bouts were defined as no engagement in locomotion, eating, drinking, or grooming for 40 s or less, while “sleep” was defined by the software as a quiet bout lasting for greater than 40 s. While this is the designation assigned by the software, there is no detailed information provided by this system about sleep quality.

Food, water, and body mass were measured by Promethion MM-1 Load Cell sensors. The amount, frequency, duration, and rate at which food and water were withdrawn from the hoppers were measured and analyzed. The body mass monitors were plastic tubes that also functioned as in-cage enrichment and nesting devices.

Respiratory gases were measured by the Promethion GA-3 gas-analyzer which measured water vapor, CO_2_ and O_2_ in ml/min. Energy expenditure was calculated using the Weir equation (52): kcal/h = 60 × (0.003941 × V̇O_2_ + 0.001106 × V̇CO_2_). Respiratory Exchange Ratio (RER) was calculated as the ratio of V̇CO_2_/V̇O_2_ where a RER of about 0.7 indicates pure lipid utilization and a RER of about 1.0 indicates pure carbohydrate utilization. Data acquisition and system control were coordinated using MetaScreen v. 2.2.8, and the obtained raw data were processed using ExpeData v. 1.9.14 (Sable Systems) and Universal Macro Collection v. 10.1.11.

### Homeostatic Model Assessment of Insulin Resistance (HOMA-IR)

Animals were fasted with water only for six hours prior glucose testing to obtain fasting measurements. Trunk blood was used to obtain blood glucose measurements on a Contour One blood glucose meter strip (Ascensia, Parsippany, NJ). Blood was then collected and centrifuged at 12,000 rpm to draw off serum for fasting insulin ELISA. Serum insulin levels were determined by an insulin ELISA assay (Crystal Chem, Downers Grove, IL). Insulin resistance was assessed by HOMA-IR score {HOMA-IR = [26 * serum insulin (ng/ml) * blood glucose (mg/dL)]/405}.

### *Listeria*-Specific T Cell Enumeration

Bone Marrow derived Dendritic Cells (BMDCs) infected with *Listeria* were used as antigen presenting cells in the T cell activation assay. BMDC were generated as previously described (53). Briefly, bone marrow was harvested from 8–12-week old C57BL/6 mice and cultured in the presence of 10 µg/ml recombinant Granulocyte-Macrophage Colony Stimulating Factor (GM-CSF) BMDCs were infected with *Lm*-10403s at a MOI of 1, and after 1 h, 20 μg/ml gentamicin (VWR) was added to the culture to inhibit bacterial replication (this concentration of gentamycin inhibits both extracellular and intracellular bacterial growth in our system, data not shown). The cells were incubated for 24 h at 37°C with 5% CO_2_ to allow antigen processing and presentation before co-culture with T cells. Splenocytes from infected or uninfected mice were cocultured with DCs at a ratio of 10:1 for 5 h in the presence of GolgiStop (monensin) (BD Biosciences). Cells were washed in FACS buffer (PBS supplemented with 3% FBS) and were incubated with AF488-anti-CD3 at 4°C for 10 min. The cells were washed in FACS buffer twice then fixed and permeabilized (BD CytoFix/CytoPerm) by incubating for 20 min at 4°C. The cells were washed in Perm/Wash buffer and incubated with PE-anti-IFN-γ for 30 min at 4°C. Cells were washed twice in Perm/Wash buffer and resuspended in FACS buffer before flow cytometric analysis.

### Glucose Transporter-1 Detection

Splenocytes isolated at indicated times post infection were washed twice in FACS buffer and were incubated with PE-Cy7-anti-Ly6C, PE-Anti Ly6G, AF488-anti-CD3, and AF647-anti-Glut-1 at 4°C for 10 min. Cells were washed twice in FACS buffer and resuspended in FACS buffer before flow cytometric analysis. The cell-associated fluorescence was measured by flow cytometry using a BD Accuri™ C6 flow cytometer and analyzed using FlowJo^®^ software.

### NBDG Uptake as a Surrogate of Cellular Glucose Uptake

The fluorescently-labeled glucose analog, 2-N-(7-nitro-benz-2-oxa-1, 3-diazol-4-yl) amino)-2 deoxyglucose, (2-NBDG) (VWR) was used as a proxy of glucose uptake. Splenocytes were washed twice in RPMI-1640 medium and treated with 2-NBDG at 37°C with 5% CO_2_ for 30 min. Cells were washed twice in FACS buffer, incubated with PeCy-7 anti-CD11b at 4°C for 10 min, washed twice in FACS buffer and resuspended in FACS buffer for cytometric analysis. The cell-associated fluorescence was measured by flow cytometry using a BD Accuri™ C6 flow cytometer and analyzed using FlowJo^®^ software. Cells were classified as NBDG (+) or (-) relative to controls.

### Statistical Analysis

Statistical analyses were performed using Prism Software, version 8 (GraphPad). Results are presented as mean +/- SD, and significance was determined using a one-way ANOVA followed by a Dunnett’s *post hoc* test. Asterisks denote level of statistical significance (*p < 0.05, **p < 0.01, ***p < 0.005, and ****p < 0.001). Linear regressions were calculated in R studio. The regression of Activity and VO_2_ included the variables Group. Control and High Dose activity and VO_2_ were used to generate the graphs in Prism Software, Version 8 (GraphPad).

### Antibodies

**Table:** 

Antibodies, (dilutions used for experiments)	Source	Identifier
PE-Cy7-anti-Ly6C (1:100)	BioLegend	Catalog: 128018 Clone: HK1.4
PE-anti-Ly6G (1:100)	BD Biosciences	Catalog:551461 Clone: 1A8
AF488-anti-CD3 (1:100)	BD Biosciences	Catalog: 557666 Clone: 145-2C11
AF647-anti-Glut-1 (1:100)	Novus Biologicals	Catalog: NB110-39113AF647
PECy7-anti-CD11b (1:100)	BioLegend	Catalog: 101216 Clone: M1/70
PE-anti-IFN-γ (1:100)	BioLegend	Catalog: 505808 Clone: XMG1.2

## Results

### Impact of Infection on Metabolic Phenotype

To evaluate the impact of bacterial infection and immune response on the systemic metabolic phenotype of the host, we infected mice with one of two doses of *Lm* and compared their metabolic phenotype to that of uninfected mice. Previous studies and our own pilot studies have determined that a bacterial dose of 2.5x10^4^ CFU/mouse induces a moderate illness that resolves within 4–5 days, ultimately conferring protective immunity (23, 24), while a lower dose (10^4^ CFU/mouse) also confers protective immunity, but without overt signs of illness. We will refer to these two infectious doses as “high dose” and “low dose” throughout the study. We used a wild type strain of *Listeria monocytogenes*, 10403s, and monitored the mice for 12 days in Promethion^®^ metabolic cages to assess multiple physiological and behavioral parameters, collectively known as metabolic phenotype. These parameters included body mass, activity, sleep, VO_2_, VCO_2_, Respiratory Exchange Rate (RER), and Energy Expenditure (EE). The metabolic data were averaged for each group of five (individually housed) mice over each 12-h period, corresponding to either the dark cycle (active period), or the light cycle (inactive period).

**Figure 1:**
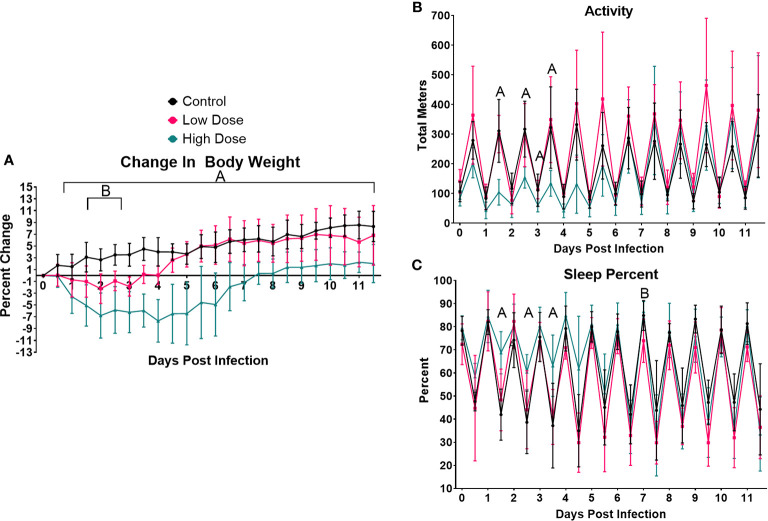
Infection-induced weight loss and lethargy.**** Analysis of change in **(A)** body weight **(B)** activity, or **(C)** sleep over the 12-day experimental period. For each parameter, the values were averaged for each individual mouse over the 12-h light/dark cycle. Each data point represents the combined average of the five animal group over a 12-h light/dark cycle. Significance was assessed using one-way ANOVA followed by a Dunnett Test for multiple comparison. Data are represented as mean ± SD. A indicates a significant between High Dose - Control and B indicates a difference between Low Dose - Control at a *p* =/< 0.05. Control (n = 6), High Dose (n = 5), Low Dose (n = 5).

We first examined changes in body mass induced by infection. Mice infected with the higher bacterial dose lost significant weight beginning at day 1 and continued to lose weight until day 4, ultimately losing a total of ~11% of their body mass (**Figure 1A**). Mice infected with the higher dose of *Lm* did not recover to their initial weight until day 7.5 and even then, gained weight more slowly than their uninfected and lower-dose infected counterparts. In fact, mice infected with the higher dose of *Lm* demonstrated significant differences in weight vs. uninfected mice from days 1 through 12 (**Figure 1A**). The duration and magnitude of weight loss in the mice infected with the lower dose of *Lm* was significantly less than those infected with the higher dose. Lower dose-infected mice lost only ~2% of their body weight, with maximal loss around day 2, and recovered to their starting weight by day 3.5. The average body mass of this group was significantly different from uninfected mice only from days 2–3.5. Thus, we observed a dose-dependent loss in body mass in mice infected with *Lm* and a sustained slowing of body mass recovery in mice infected with the higher dose.

The next parameters examined were activity and sleep (**Figures 1B, C**). Activity was measured in total meters, averaged across each treatment group over each 12 h period. As expected, uninfected mice were much more active during the dark cycle than the light, establishing a baseline level and pattern of activity. We also observed no differences in activity at any time point between uninfected control animals and mice infected with the lower dose of *Lm* (**Figure 1B**). However, there was a marked reduction in the activity of mice infected with the higher dose of *Lm* (~40–50% of control) during the active cycles of days 1, 2, and 3 as well as in the inactive cycle of day 4. However, activity returned to control levels by the active cycle of day 5 in these mice (**Figure 1B**).

Mice infected with the higher dose of bacteria also exhibited increased time spent in quiet/sleep compared to control mice (**Figure 1C**). The periods of significantly increased sleep in the high dose group overlapped almost completely with the periods of decreased activity in this group during the active periods of days 1, 2, 3, and 4, but returned to control levels of sleep by day 5 (**Figures 1B, C**). Again, no significant differences were observed between uninfected and low dose infected mice in sleep at any time point (**Figure 1C**). Thus, in addition to weight loss, mice infected with the higher dose of *Lm* experienced markedly reduced activity and more time spent in sleep, typical symptoms of illness or sickness behavior (54, 55). However, even though the lower dose of *Listeria* is known to induce a strong T cell response (**Figure 5**) and protective immunity (22, 23, 32), this level of infection induced no detectable changes in activity or sleep at any time.

**Figure 2:**
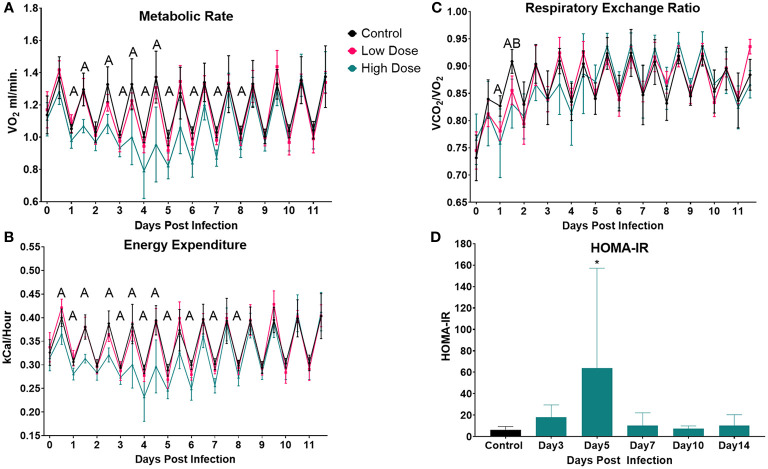
Infection-induced changes to systemic metabolism. Analysis of change in **(A)** Metabolic rate (VO_2_), **(B)** Energy Expenditure, or **(C)** Respiratory Exchange Ratio (VCO_2_/VO_2_) over the 12-day experimental period. Each data point represents the average for a 12-h light/dark cycle. The Weir Equation was used to calculate EE (kcal/h = 60 × (0.003941 × V̇O_2_ + 0.001106 × V̇CO_2_), and an ANCOVA was utilized to adjust EE for bodyweight. Significance was assessed using one-way ANOVA followed by a Dunnett Test for multiple comparison. Data are represented as mean ± SD. A indicates a significant between High Dose - Control and B indicates a difference between Low Dose - Control at a *p* =/< 0.05. Control (n = 6), High Dose (n = 5), Low Dose (n = 5). **(D)** The HOMA-IR was used to determine insulin resistance in mice infected the high dose at various time points across the experiment. *p < 0.05.

We next wanted to determine how infection impacted systemic metabolic parameters including the exchange rate of O_2_, which is often used as a proxy of metabolic rate (**Figure 2**). We observed a significant drop in VO_2_ in the high dose-infected group vs. control beginning during the active period of day 1 and continuing through the active period of day 4 (**Figure 2A**). Interestingly, a decrease in VO_2_ in the high dose group were observed even during several inactive periods between days 3 and 8. Notably, there were no significant difference in VO_2_ at any time point between mice infected with the lower dose of *Lm* and control mice (**Figure 2A**).

To determine if infection and immune response altered carbon substrate utilization and/or overall energy expenditure, we calculated the Respiratory Exchange Rate (RER, VCO_2_/VO_2_) and energy expenditure (using the Weir equation, adjusted for body mass) (**Figures 2B, C**). RER was significantly reduced in mice infected with the high dose of *Lm* from day 1.5 to day 2, indicating a shift toward increased lipid utilization during this time (**Figure 2C**). A similar shift toward enhanced lipid utilization was also observed in the low-dose infected mice, but only at day 2. There was also a significant decrease in overall energy expenditure in mice infected with the higher *Lm* dose, beginning on day 0.5 and continuing through day 8. This reduced energy expenditure was significant during both active and inactive periods (**Figure 2B**). There were no significant differences in energy expenditure between mice infected with low dose *Lm* and uninfected controls (**Figure 2B**).

Our final measure of systemic metabolism was insulin resistance, which has been observed in a variety of infections (56, 57). Using the Homeostatic Model Assessment of Insulin Resistance (HOMA-IR), we observed a spike in IR that was significant at day 5 in the mice infected with the high dose and a similar trend at day 3 which was not significant (**Figure 2D**).

### Metabolic Rate vs. Activity

Infection and sickness have been reported to raise resting metabolic rate (RMR) in numerous studies (4, 58–60). However, our results demonstrated that infection led to a decrease in VO_2_ and energy expenditure. One explanation for this difference might be the greatly decreased activity observed in the high dose group in our system. Thus, we sought to examine the relationship between activity and VO_2_ in the high dose-infected and uninfected animals using a linear regression model. Since the most pronounced differences in systemic metabolic profile between infected and uninfected groups occurred during the active cycle (night) we used these values to examine the relationship between activity and VO_2_.

**Figure 3:**
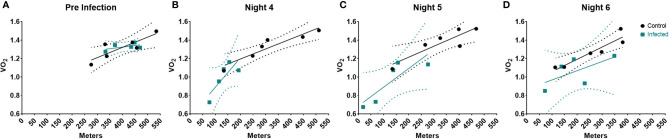
Linear Regression of Activity and VO_2_ during the course of the infection. Linear Regression at **(A)** Pre-infection, **(B)** Night 4, **(C)** Night 5, & **(D)** Night 6 post infection. Dotted regions represent the 95% Confidence interval. Control (n = 6), Infected (n = 5).

Prior to infection, there were no significant differences between groups (**Figure 3A** and **Supplementary Table I** Pre-infection). However, by night 4, we observed significant decreases in metabolic rate and activity in the infected animals (**Figure 3B**), resulting in a significant difference between the two groups (**Supplementary Table I**). By night 5, the relationship between systemic metabolic rate and activity between control and infected mice began to resolve; and we no longer observed a group effect (**Figure 3C** and **Supplementary Table I**). Finally, by night 6 the metabolic rate of infected mice returned to control level, and again there was no group effect (**Figure 3D** and **Supplementary Table I**). Thus, this analysis revealed that the relationship between activity and metabolic rate does change over the course of the infection but does not correspond to previous reports of increased metabolic rates. These results do however indicate that activity was more metabolically demanding in the infected group during the peak of sickness behavior.

### Kinetics of Bacterial Infection and T Cell Response

**Figure 4:**
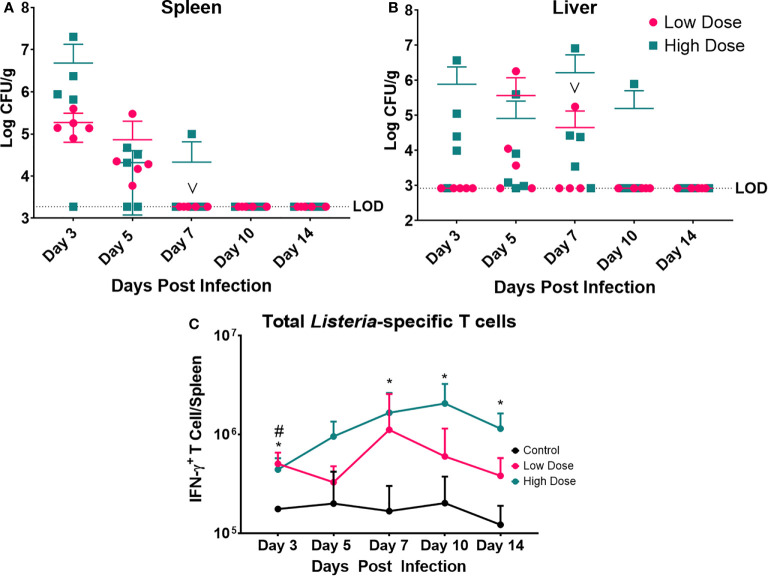
Bacterial Enumeration and *Listeria*-specific T cell response. Bacterial burden at various time points following a *Listeria* infection in the **(A)** spleen and **(B)** liver. LOD indicated limit of detection. Data are represented as mean ± SD. High Dose (n = 5), Low Dose (n = 5). **(C)** *Listeria*-specific IFN-γ producing T cells were enumerated at various time points post infection in the spleen following a *Listeria* infection. Statistical analysis was performed by a Mixed-effect analysis followed by a Dunnett Test for multiple comparison. Data are represented as mean ± SD. * indicates a significant between High Dose (n = 5) - Control (n = 3) and ^#^ indicates a difference between Low Dose (n = 5) - Control (n = 3). *p < 0.05, ^#^ *p* < 0.05. v indicates a mouse death in the day 7 post infection low dose group.

To determine how the kinetics of systemic metabolism corresponded to bacterial burden and to the anti-*Listeria* immune response, groups of conventionally housed mice were infected (simultaneously with those housed in metabolic cages), again with either 1x10^4^ CFU/ml or 2.5x10^4^ CFU/ml of *Lm-*10403s, in addition to the uninfected controls. These animals were sacrificed over the course of fourteen days post infection to monitor bacterial burden in the spleen and liver as well as the *Listeria*-specific T cell response in the spleen (**Figure 4**).

The bacterial burden was maximal for both infection groups at day 3 in the spleen and day 7 in the liver (**Figures 4A, B**). Yet, the level of infection was dramatically higher in the high dose-infected group vs. the low dose group in the spleen at day 3. The mice infected with the low dose showed no detectable bacteria in the spleen after day 5 and after day 7 in the liver. The kinetics of clearance were slightly slower for the high dose-infected group with evidence of bacterial burden at day 7 in the spleen and out to day 10, and 14 in the liver (in one animal per group). Thus, though the inoculating doses were only different by 2.5-fold, the bacterial burden in the organs was dramatically higher and took longer to clear in the high dose group. Perhaps the higher dose exceeded a threshold of control, requiring more time and perhaps more immune mechanisms for clearance.

We also examined the *Listeria*-specific T cell response to determine if the kinetics and magnitude of this response were significantly different at the two infectious doses (**Figure 4C**). Splenocytes were cultured in the presence of syngeneic *Listeria*-infected DC to measure the *Listeria*-specific T cell response in the form of IFN-γ production. Measurement of IFN-γ production was enabled by intracellular cytokine staining and flow cytometry compared to splenocytes from control (uninfected) mice. The observed peak of the *Lm*-specific T cell response was at day 7 in the low dose infected group and day 10 in the high dose group (**Figure 4C**). While the peak responses occurred on different days, and there was a trend toward higher numbers of *Lm*-specific T cells in high dose infected group. Additionally, the high dose infected group exhibited a significantly higher the number of *Lm-*specific T cells at day 3, 7, 10, and 14 post infection, while the low dose infected group only differed at day 3 post infection from control.

### Changes in Cellular Metabolic Status Upon Infection

To determine whether changes in systemic metabolic phenotype correspond to changes in immune cellular metabolism over the course of infection, we measured the expression of the glucose transporter (Glut-1) on cells expressing markers typical of a broad population of leukocytes (Ly6C), neutrophils (Ly6G), and T cells (CD3). The Glut-1 transporter is up-regulated on many cell types after activation and is used as an indicator of cells shifting into a state of aerobic glycolysis (40, 44). Given that the high dose infection group displayed the most significantly different metabolic phenotype (systemic metabolism), we monitored the cellular metabolic status of this group compared to the uninfected controls.

**Figure 5:**
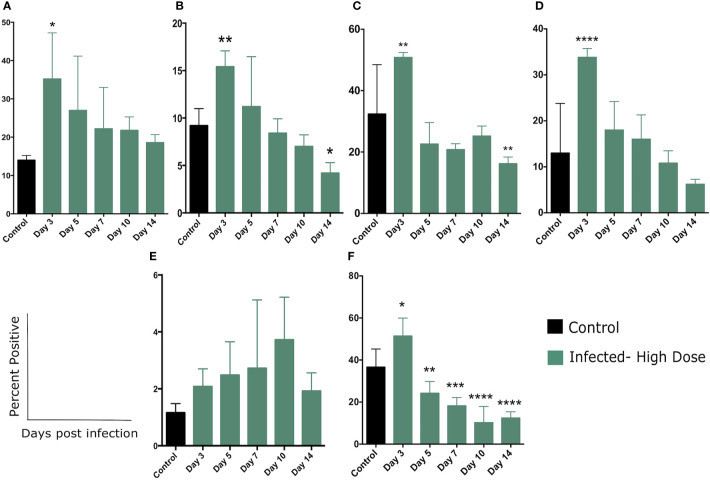
Changes in cellular metabolism in Ly6C and Ly6G-expressing cells. **(A)** Percent of splenocytes expressing Ly6C following *Lm* infection and **(B)** Percent of Ly6C^+^ cells expressing Glut1 over the course of the infection. **(C)** Percent of Ly6C^int^ or **(D)** Ly6C^hi^ cells expressing Glut1. **(E)** Percent of splenocytes expressing Ly6G following *Lm* infection and **(F)** Percent of Ly6G^+^ cells expressing Glut1 over the course of the infection. Significance was assessed using one-way ANOVA followed by a Dunnett Test for multiple comparison. Data are represented as mean ± SD. High Dose (n = 5) - Control (n = 3). *p < 0.05, **p < 0.01, ***p < 0.005, ****p < 0.0001.

There was a significant increase in the percentage of Ly6C^+^ cells in the spleen of high dose-infected mice at day 3 post infection (**Figure 5A**, **Supplementary Figure 3A**), consistent with previous studies (20, 21, 29). Ly6C is expressed at different levels by a number of cell types including monocytes, neutrophils, and lymphocytes at various stages of activation. The gating strategy for these experiments is detailed in **Supplementary Figure 1** and sample primary data is included in **Supplementary Figure 3A**. These flow cytometric plots illustrate the increase in Ly6C^+^ cells in the spleen of high dose-infected mice as well as the increased expression in Glut-1 by these cells. We next quantitated the expression of the glucose transporter Glut1 on the total Ly6C^+^ cells (**Figure 5B**). The percentage of Ly6C^+^ cells expressing Glut-1 peaked on day 3 (and was significant at this timepoint), decreased on day 5, and returned to control level on day 7 post infection, declining through day 14 (**Figure 5B**).

We noted that within the Ly6C^+^ cells, there were also distinct populations expressing either a high level of Ly6C (Ly6C^hi,^a level of expression typical of inflammatory or activated monocytes as well as activated lymphocytes) or an intermediate level (Ly6C^int^, associated with several polymorphonuclear cell types) (**Supplementary Figures 3A, B**). Over the course of the infection we observed an increase in the percent of the Ly6C^+^ population that were Ly6C^hi^, with significant differences from control at days 7, 10, and 14 (**Supplementary Figure 3B**). The percent of both the Ly6C^int^ and Ly6C^hi^ populations expressing Glut1 increased significantly at day 3 (**Figures 5C, D**, respectively) and declined through the rest of the experiment. The most significant difference was observed in the Ly6C^hi^ population in which a high proportion of these cells were Glut1^+^ at day 3 (**Figure 5D**). This finding is suggestive of a metabolic shift in activated/inflammatory monocytes as well as activated lymphocytes during *Lm* infection.

We also examined the expression of Glut1 by cells expressing Ly6G, a common marker of neutrophils. In contrast to Ly6C expression, we did not observe a significant increase in this population in the spleen, but there was a steady trend of increase throughout the time frame of the experiment (**Figure 5E** and **Supplementary Figure 4A**). The flow cytometric plots in supplemental figure 4A illustrate the increase in Ly6G^+^ cells in the spleen of high dose-infected mice at day 3 as well as a modest increase in expression of Glut-1 by these cells. While neutrophils are known to play an important role in *Lm* clearance, we may have missed the peak of these cells in the days prior to our first sampling at day 3. We did however, note a significant increase in the percent of Ly6G^+^ cells expressing Glut1 at day 3 and a return to control (and below) levels by day 5 (**Figure 5F**). Taken together, we observed a significant increase in the proportion of both Ly6C and L6G-expressing cells that also expressed Glut1, primarily peaking at day 3 post infection (**Figure 5**). This change in metabolic status of several leukocyte types coincides with the majority of changes in systemic metabolic phenotype.

To further examine the shift in cellular metabolism of myeloid cells, we measured glucose uptake by CD11b^+^ cells though the use of a fluorescent glucose analog 2-(N-(7-Nitrobenz-2-oxa-1,3-diazol-4-yl)Amino)-2-Deoxyglucose (2-NBDG). We observed a modest uptake of 2-NBDG by a small percentage of CD11b^+^ cells which was significantly increased on day 7 and returned to control level by day 10 (**Supplementary Figure 4B**). Taken together, these findings highlight the recruitment of inflammatory cells and neutrophils cells to the spleen during *Lm* infection and demonstrate that a portion of these cells utilize aerobic glycolysis during this response, based on an increase in glucose transporter expression and increased glucose uptake (**Figure 5**).

**Figure 6:**
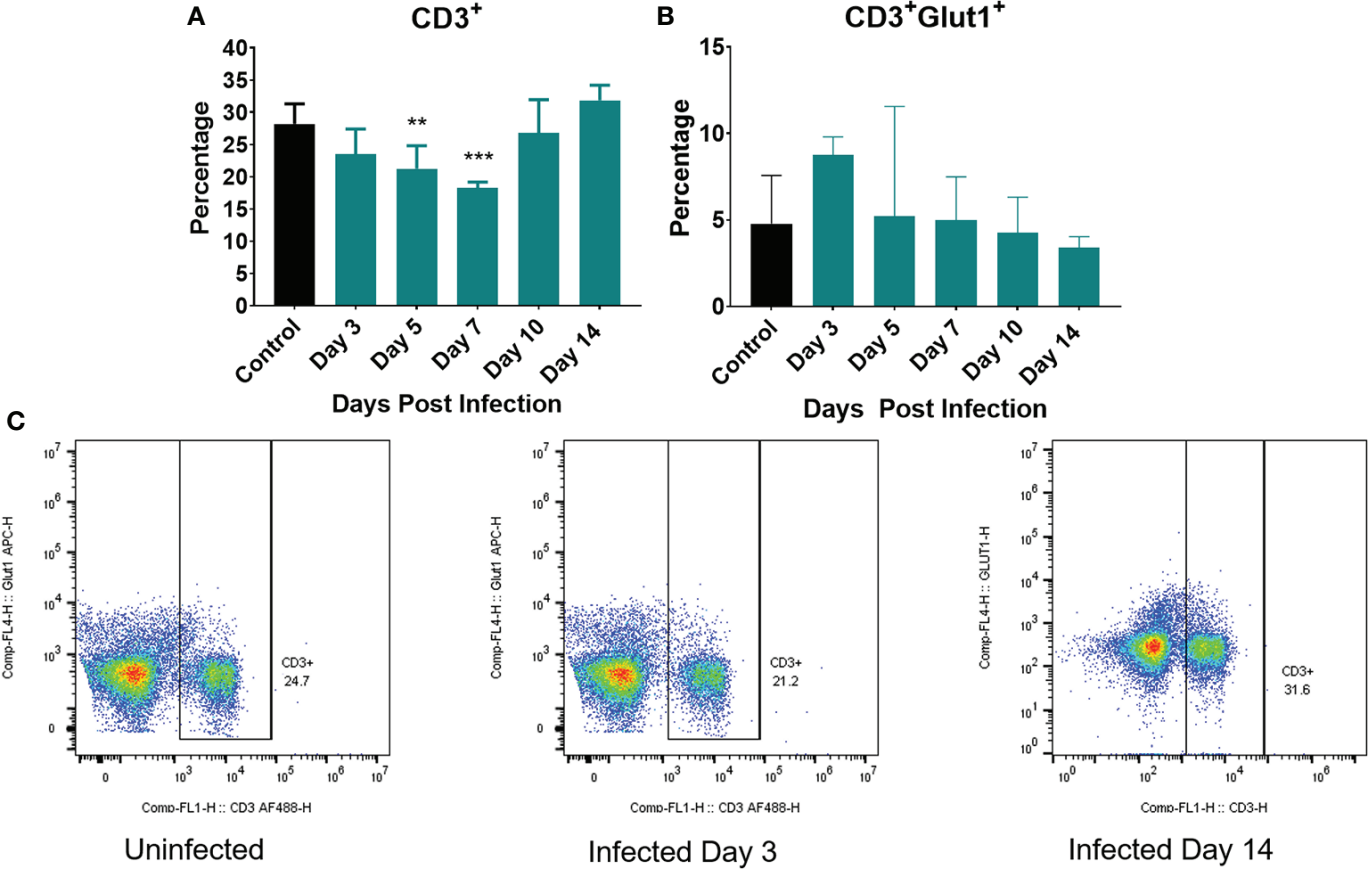
Markers of cellular metabolism of T cells. Percent of splenocytes expressing **(A)** CD3^+^ (T cells) and **(B)** percent of CD3+ cells expressing Glut1 over the course of the infection. **(C)** Representative flow cytometric plots of CD3 and Glut1 on T cells on uninfected controls and at day 3 and 14 post infection. Significance was assessed using one-way ANOVA followed by a Dunnett Test for multiple comparison. Data are represented as mean ± SD. High Dose (n = 5) - Control (n = 3). *p < 0.05, **p < 0.01, ***p < 0.005.

We next examined the metabolic status of T cells by measuring expression of Glut1 on CD3^+^ cells (**Figure 6**). **Figure 6A** depicts the percentage of T cells (CD3^+^) in the spleen over the course of the infection (total T cells). We initially observed a decrease in the percentage of T cells at day 3 continuing until day 7 post infection (**Figure 6A**). This early T cell decline has been previously observed as *Listeria* infection is known to induce T cell apoptosis (61, 62). By Day 10 post infection, the percentage of total T cells began to increase and returned to control levels (**Figure 6A**). **Figure 6C** depicts the expression of Glut-1 on CD3^+^ T cells while **Figure 6B** is representative of several compiled, independent measurements of these parameters. The gating strategy for these experiments is detailed in **Supplementary Figure 2** as well as they overall number of T cells per organ. We observed little change in the number of T cells expressing Glut-1 and only a modest increase in Glut-1 expression on T cells over the course of the infection, none of which were significantly different when compared to control (**Figure 6B**).

## Discussion

To our knowledge, ours is the first study to simultaneously monitor the systemic and cellular metabolic phenotype in a live infection in concert with measurements of immune response and bacterial burden. Our use of both low and high bacterial burdens also allowed us to discern differences associated with sickness behavior (high dose) vs. an immunization model/low level infection that does not induce overt illness (low dose). The longitudinal analysis of metabolic phenotype over the entire course of infection and primary immune response also provided a detailed assessment of the metabolic changes when compared to previous studies in which metabolic rates were measured at isolated time points (4, 59, 60). These combined analyses provided new insights into the respective demands associated with the timing of innate and adaptive immunity and potential trade-offs with other life history traits.

**Figure 7:**
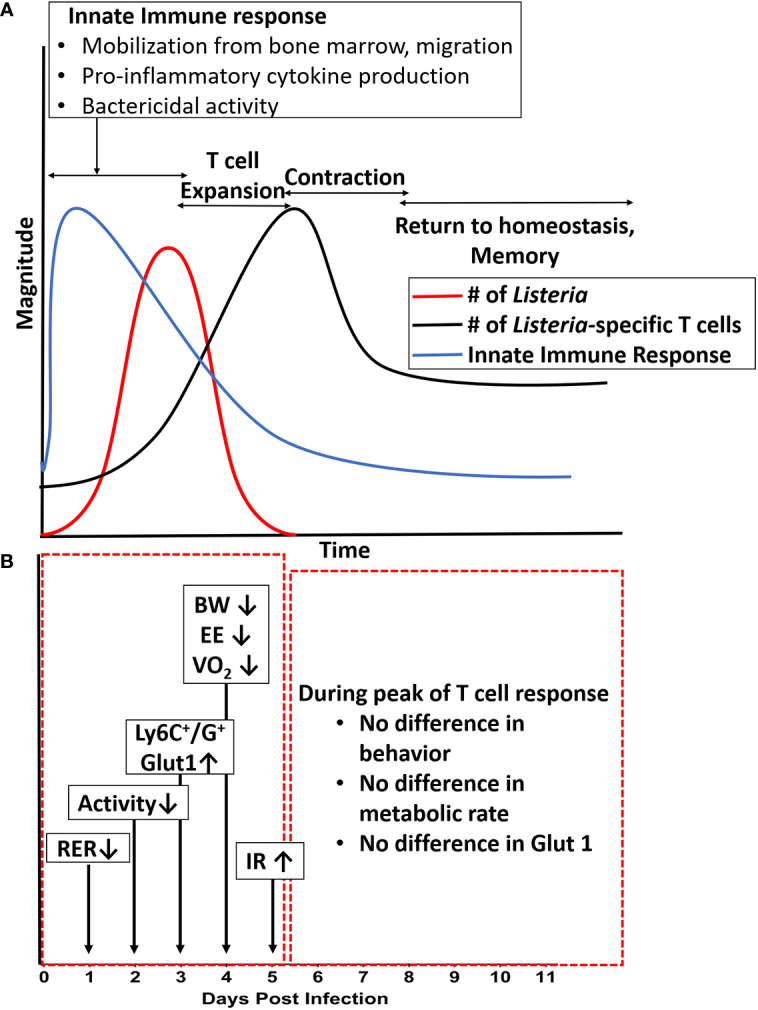
Summary of metabolic changes against the backdrop of immune response over the course of a primary infection. **(A)** Illustration of the magnitude of bacterial infection, innate response, and adaptive (T cell) response over time. **(B)** Summary of changes in systemic and cellular metabolic phenotype over time. Long arrows denote days at which most significant differences were observed in specific readout vs. uninfected control. Shorter arrows indicate direction of change in a specific readout. BW, body weight; EE, energy expenditure; HOMA-IR, Homeostatic Model of Insulin Resistance; RER, Respiratory Exchange Ratio; VO_2_, metabolic rate.

The plots in **Figure 7** summarize and collectively illustrate our metabolic findings on the backdrop of infection, immune response, and clearance. The diagram in **Figure 7**, panel A summarizes the well-established kinetics of bacterial infection, innate immune response, and expansion and contraction of the T cell response over the first eleven days following intraperitoneal infection with *Listeria* (16–19), which were confirmed in our study. In the first five days following infection, bacterial burden (red line), peaks around day 3–5 and by day 7, bacteria are mostly cleared depending on the inoculating dose (23, 24) and (**Figure 4**). During this same timeframe, the innate immune response (blue line) kicks in within the first hours and lasts throughout the first 3–5 days with mobilization of neutrophils and monocytes from the bone marrow and production of IL-1, IL-6 and TNF-α (25–31) and (**Figure 5A**). On the heels of the innate response, the adaptive (T cell) response (Green line) becomes detectable around day 5 and reaches maximal or peak proliferation around 7–10 days (22, 23, 32, 33) and (**Figure 4C**).

The diagram in panel B of **Figure 7** pinpoints the times at which the systemic and cellular metabolic phenotype were the most significantly different from control. In the red box encompassing days 1–5, we see that several aspects of the metabolic phenotype are altered in the animals infected with the high dose. First, at day 1–2, we observed a decrease in RER (Respiratory Exchange Rate), indicating a preferential use of lipids as carbon source. At day 2, we began to see decreased activity which lasted through day 4. At days 3–5, we observed increased expression of Glut-1 and glucose uptake by Ly6C^+^ and Ly6G^+^ cells (likely monocytes, activated lymphocytes, and neutrophils) as well as systemic insulin resistance, and at day 4 we observed the peak decreases in weight (BW), energy expenditure (EE), and metabolic rate (VO_2_). Thus, our findings lead us to conclude that for systemic *Lm* infection, significant metabolic changes at the systemic and cellular levels coincided with the timing of maximal innate response. Given that this metabolic demand was associated with decreased activity and energy expenditure, our study illustrates an important life history trade-off between infection resistance and activity. These findings also call into question the relative roles of the innate immune response (and specific components therein) and that of bacteria-induced damage and pathogenesis in sickness behavior and metabolic demand. These are experimental questions we are currently pursuing. Further, given the limited number of fluorescent channels available in our flow cytometric system, future studies will include a more detailed, multi-parameter analysis to better define the immunometabolic phenotype of the specific cell types responding to the infection.

In contrast, during the period indicated by the red box on the right (days 5–11, **Figure 7**, panel B), we observed no differences between infected and control animals in activity, energy expenditure, RER, expression of Glut-1 on monocytes, or other measures of systemic or cellular metabolic phenotype. Thus, during this period of maximal expansion of the adaptive immune response, the host exhibited no detectable metabolic burden or tradeoffs. Likewise, at the lower infectious burden, though a protective T cell response (data not shown) was observed (**Figure 4C**), little to no tradeoffs occurred (**Figures 1**, **2**). Thus, only specific aspects of the immune response (likely innate) appear to require such tradeoffs.

**Figure 8:**
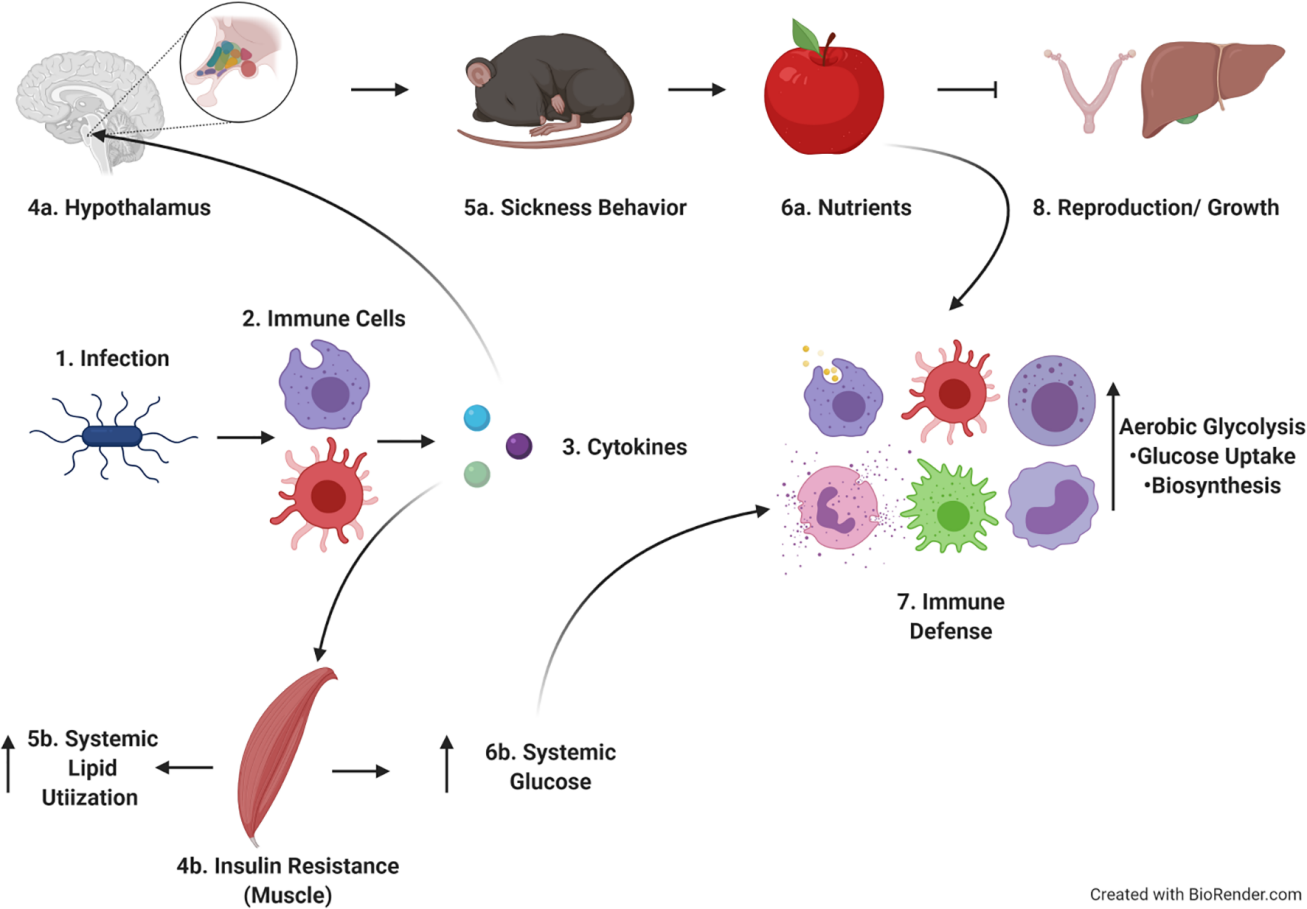
Hypothetical model of findings within the context of life history theory. Upon infection, immune cells produce cytokines which act on both brain and muscle. The effects on the brain lead to sickness behavior and cause tradeoffs with activity, reproduction and growth. Cytokines acting on muscle leads to insulin resistance and increased systemic lipid utilization, allowing increased circulating glucose to be used by immune cells as they proliferate and develop effector function. Image created in Biorender.com.

Applying a Life History Theory framework to our studies also helps to tie together our findings into a cohesive model (**Figure 8**). Following infection, cells of the innate immune system such as neutrophils, monocytes, and macrophages respond rapidly to bacterial PAMPs with the production of pro-inflammatory cytokines such as IL-1, IL-6, and TNF-α (25–31). These cytokines act systemically on a variety of tissues, inducing specific responses. For example, pro-inflammatory cytokines induce the production of lipid mediators such as prostaglandins. Prostaglandins act on specific nuclei within the hypothalamus to induce fever, reduce activity, and limit appetite (5, 63–66). These changes lead to sickness behavior and tradeoffs between immune response and activity, (and by extension likely reproduction and growth, though those possibilities need to be more extensively tested in our system). Pro-inflammatory cytokines such as TNF-α also act on skeletal muscle and liver increasing blood glucose and insulin resistance. One potential explanation for how these observations fit together is that infection-induced insulin resistance could drive preferential utilization of lipids systemically, making glucose available for utilization by immune cells (which express higher levels of Glut-1 and consume more glucose, **Figure 5**). This hypothesis has also been posed by Wang, et al. (67).

There is ample evidence from studies *in vitro*, demonstrating that when cells of the innate immune system become activated, they exhibit a shift in cellular metabolic pathways away from oxidative phosphorylation (OXPHOS) toward aerobic glycolysis. This shift is thought to allow for the increased demand of biosynthesis (cytokine production) (37, 38). Our study confirms and extends these previous findings *in vitro* with *in vivo* evidence of the same shift. The previous reports focused on these changes only up to hours after pathogen recognition receptor activation.

We also observed a *Listeria*-specific T cell response in both low and high dose-infected mice which peaked at day 7 or 10, respectively (**Figure 4C**). Notably, while T cells have been reported to undergo a shift to aerobic glycolysis *in vitro* (68), we did not observe significant Glut-1 upregulation on T cells at any timepoint (**Figure 6B**). Perhaps the *Lm*-induced apoptosis of T cells during the early stages of infection inhibited the expression of Glut-1 and/or the shift to this metabolic pathway (61, 62). Alternatively, a metabolic shift in T cell metabolism might be more detectable in a secondary expansion to re-infection in which many more T cells would be undergoing expansion. This is also the focus of ongoing investigation.

Infection has been shown to raise resting metabolic rate (RMR) which is an animal’s metabolic rate while resting and fasting (4, 58–60). However, many studies reporting RMR had limitations such as animal restraint (minimizing movement and potentially causing stress) (4, 58–60). We were unable to directly measure RMR in our study because the metabolic cages serve as conventional housing, allowing for locomotion. However, monitoring EE during the 30 min with the lowest activity score over the 12-h cycle is a close correlate to RMR. When we compare this “low activity EE” measured in our system, it followed the same pattern as average daily EE (data not shown, **Figure 2B**). We were also able to monitor average daily metabolic rate which is defined as the metabolic rate of a free-living animal that may or may not be in a thermoneutral zone (69, 70). This is likely a better parameter for comparison, since it resembles an animal’s natural environment by allowing for activity. In contrast to previous studies, we observed a decrease in metabolic rate during infection (**Figure 2A**).

To address the discrepancy between ours and previous studies, we wanted to better understand the relationship between activity and metabolic rate over the course of infection using a linear regression model. We observed that the relationship between activity and metabolic rate differed over the course of the infection (**Figure 3**). During the period with greatest differences in metabolic phenotype (VO_2_, bodyweight, activity & sleep), we observed an increase in the slope for infected mice (**Fig. 3B**). Thus, for each additional step taken by infected mice, their metabolic rate increased more than control. We therefore postulate that the reduced metabolic rates we observed under high dose infection conditions were likely primarily driven by decreased activity, which would not have been captured in previous approaches. This highlights the importance of decreased activity in shaping the presentation of sickness behavior (4, 71). Thus, our findings are consistent with the established hypothesis that immunity does induce trade-offs, particularly with activity and potentially growth (**Figure 1**) (1, 2). Our study also extends previous knowledge by demonstrating that the time in which trade-offs are observed coincides with the innate response, not with expansion of adaptive immunity.

Infectious doses of *Listeria* in the range of those used in our study are known to induce protective immunity in mice (23, 24). While both infectious doses induce protective immunity from future *Listeria* infection, the high dose of *Listeria* also yielded sickness behavior and a hypometabolic state in infected animals. Thus, our study illustrates an important phenomenon, that the threshold of infection/exposure required to induce protective immunity is below that required to induce trade-offs between growth, reproduction, and maintenance. This phenomenon is used to great advantage in vaccination. While there are notable side effects to several vaccines driven by the innate immune system (72–75), they ideally provide protective immunity through the development of strong adaptive immune responses with limited disease symptoms including sickness behavior. While further investigation is required, our findings collectively support the hypothesis that the innate immune response to *Listeria monocytogenes* is a stronger driver of life history trade-offs than the adaptive.

## Data Availability Statement

The data sets presented in this article are not readily available. Requests to access the datasets should be directed to ehs0009@auburn.edu.

## Ethics Statement

The animal study was reviewed and approved by Auburn University Animal Care and Use Committee.

## Author Contributions

RJ completed all experiments, optimized experimental design, and completed data analysis and wrote manuscript. AO and LW assisted with completion of experiments, data analysis, and edited manuscript. MG provided training and oversight of metabolic cage experiments and assisted with metabolic data measurement and analysis. EH designed experiments, provided oversight, and edited manuscript. All authors contributed to the article and approved the submitted version.

## Funding

These studies were supported by internal grant funds from Auburn University, a HATCH award from USDA/Alabama experiment station, and support from the College of Sciences and Mathematics and Department of Biological Sciences at Auburn University.

## Conflict of Interest

The authors declare that the research was conducted in the absence of any commercial or financial relationships that could be construed as a potential conflict of interest.

## Supplementary Material

Supplementary Figure 1 Gating strategy for Ly6C flow cytometric analysis. **(A)** Live cell gate created based on forward and side scatter. **(B)** Live cells were analyzed for doublet exclusion based on forward scatter height vs. forward scatter area. Cells within the indicated gate were further analyzed for **(C)** Ly6C and Glut1 expression. Ly6C+ cells were included based on gate set on negative control and **(D)** were further divided based on level of Ly6C staining into Ly6C^hi^ and Ly6C^int^. **(E)** Glut1 staining on Ly6C^int^ and **(F)** Ly6C^hi^ cells based on control gates.

Supplementary Figure 2 Total CD3+ cell counts and gating strategy for CD3 analysis. **(A)** Number of CD3+ cells per spleen. **(B)** Number of CD3+ cells expressing Glut1. **(C)** Gating strategy indicating cells selected for live gate, doublet exclusion, CD3+ staining and Glut1 expression on CD3+ cells.

Supplementary Figure 3 Sample Ly6C and Glut1 expression on splenocytes from uninfected, day 3 and day 14 post infection. **(A)** Detailed plots and gated analysis of Ly6C (int and hi) staining on the x axes and Glut1 staining on the Y axes. **(B)** Proportion of Ly6C^+^ cells expressing high (Ly6C^hi^) or intermediate (Ly6C^int^) levels over time post infection.

Supplementary Figure 4 Sample Ly6G and Glut1 expression and uptake of NBDG by CD11b^+^ cells. **(A)** Flow cytometric plots of Ly6G and Glut1 expression on splenocytes from uninfected control and day 3 post infection. Gate indicates percent Ly6G +. **(B)** Uptake of glucose analog NBDG by CD11b^+^ cells over time post infection.
