# Supplementary figures and images for: The Systemic and Cellular Metabolic Phenotype of Infection and Immune Response to Listeria monocytogenes
Source: Front Immunol. 2021 Feb 8;11:614697. doi: 10.3389/fimmu.2020.614697 (PMC7897666; doi:10.3389/fimmu.2020.614697)

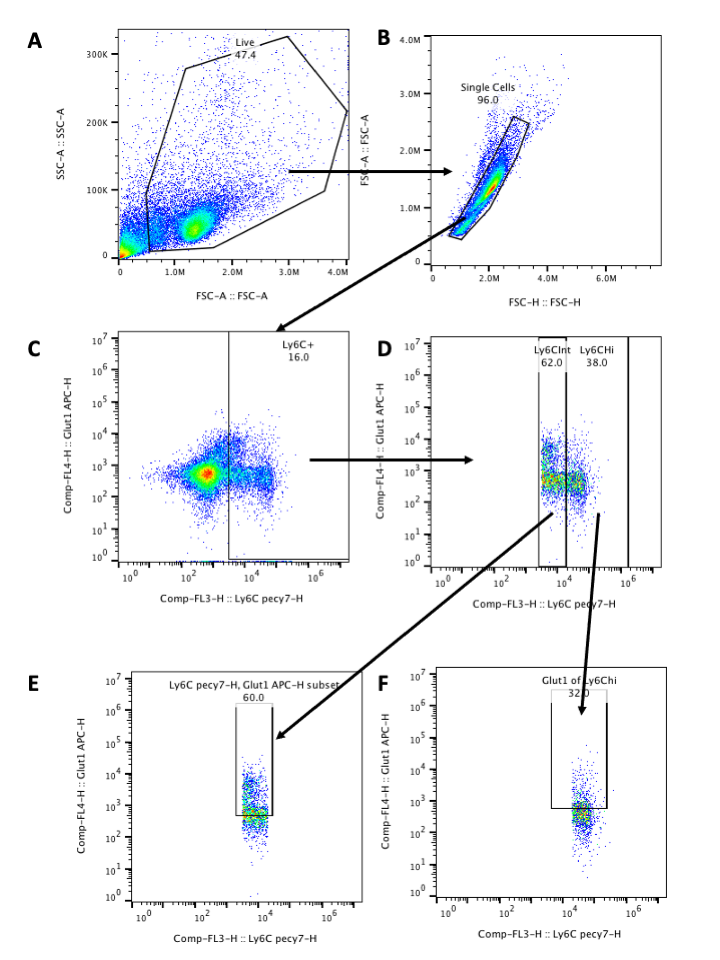

Supplement: Supplementary Figure 1 — Gating strategy for Ly6C flow cytometric analysis. (A) Live cell gate created based on forward and side scatter. (B) Live cells were analyzed for doublet exclusion based on forward scatter height vs. forward scatter area. Cells within the indicated gate were further analyzed for (C) Ly6C and Glut1 expression. Ly6C+ cells were included based on gate set on negative control and (D) were further divided based on level of Ly6C staining into Ly6Chi and Ly6Cint. (E) Glut1 staining on Ly6Cint and (F) Ly6Chi cells based on control gates. [file Image_1.tiff]

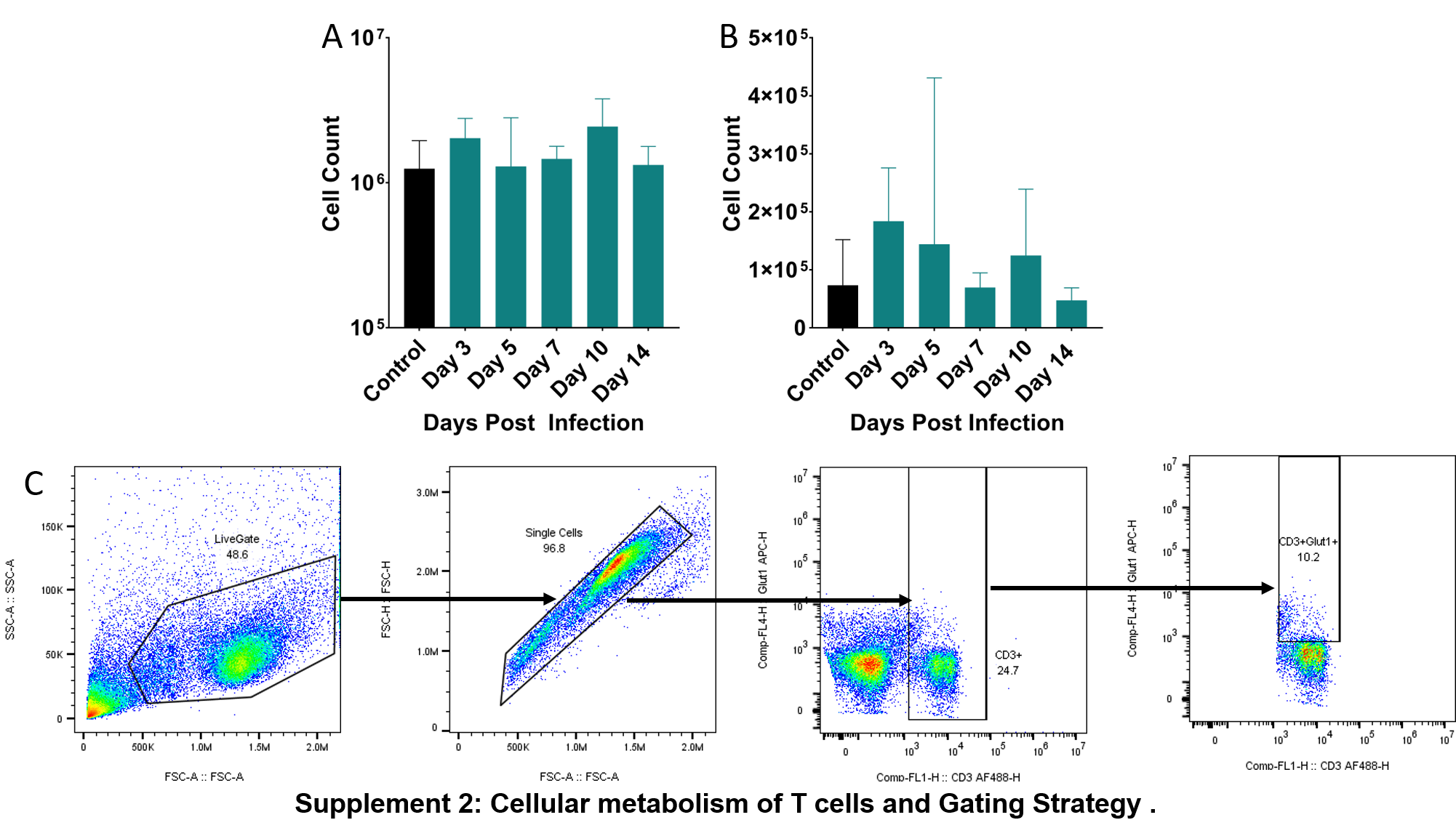

Supplement: Supplementary Figure 2 — Total CD3+ cell counts and gating strategy for CD3 analysis. (A) Number of CD3+ cells per spleen. (B) Number of CD3+ cells expressing Glut1. (C) Gating strategy indicating cells selected for live gate, doublet exclusion, CD3+ staining and Glut1 expression on CD3+ cells. [file Image_2.tif]

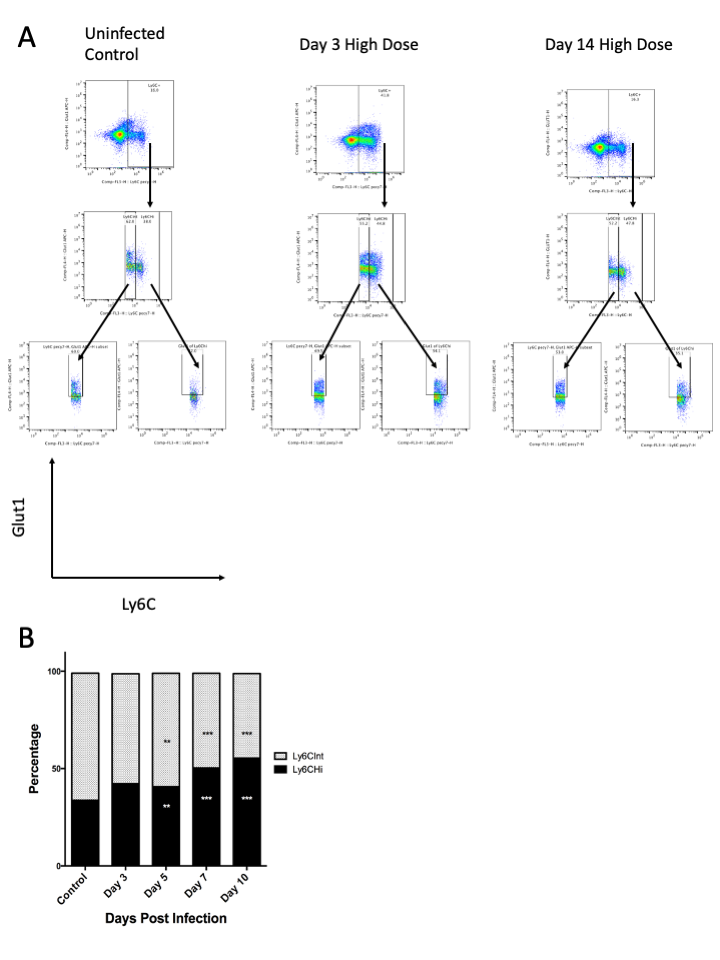

Supplement: Supplementary Figure 3 — Sample Ly6C and Glut1 expression on splenocytes from uninfected, day 3 and day 14 post infection. (A) Detailed plots and gated analysis of Ly6C (int and hi) staining on the x axes and Glut1 staining on the Y axes. (B) Proportion of Ly6C+ cells expressing high (Ly6Chi) or intermediate (Ly6Cint) levels over time post infection. [file Image_3.tiff]

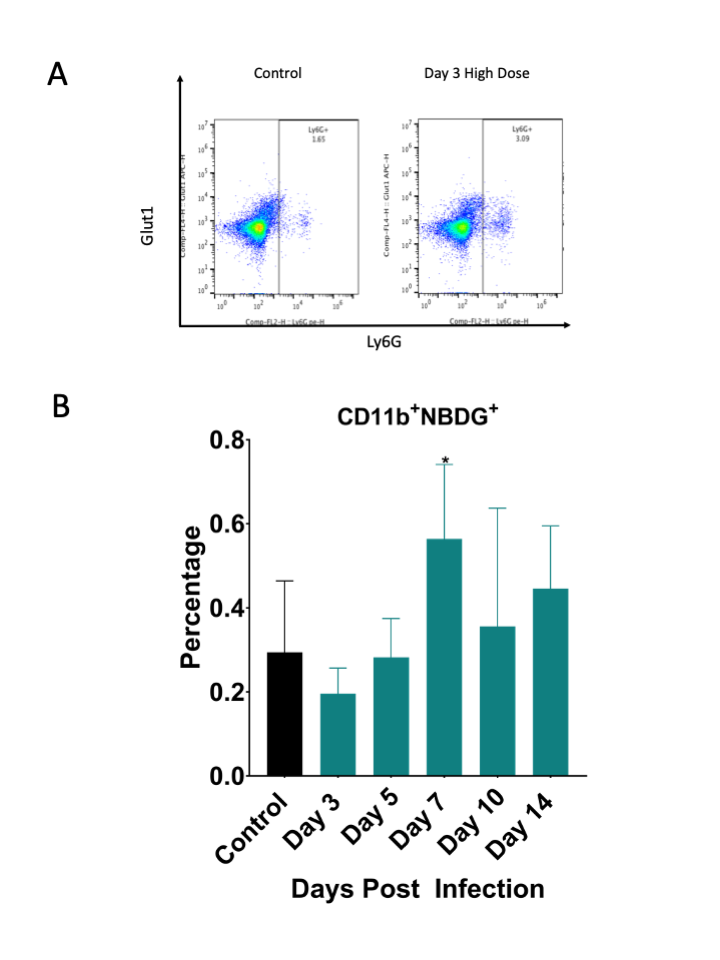

Supplement: Supplementary Figure 4 — Sample Ly6G and Glut1 expression and uptake of NBDG by CD11b+ cells. (A) Flow cytometric plots of Ly6G and Glut1 expression on splenocytes from uninfected control and day 3 post infection. Gate indicates percent Ly6G +. (B) Uptake of glucose analog NBDG by CD11b+ cells over time post infection. [file Image_4.tiff]
