# Supplementary material for: The Systemic and Cellular Metabolic Phenotype of Infection and Immune Response to Listeria monocytogenes
Source: Front Immunol. 2021 Feb 8;11:614697. doi: 10.3389/fimmu.2020.614697 (PMC7897666; doi:10.3389/fimmu.2020.614697)
Supplement: Supplementary file 5 [file Table_1.docx]

**Supplemental Table 1: Results of ANCOVA**

|  | **Pre-Infection** | | | **Night 4** | | | **Night 5** | | | **Night 6** | | |
| --- | --- | --- | --- | --- | --- | --- | --- | --- | --- | --- | --- | --- |
| Predictor Variable | Estimate | SE | p-value | Estimate | SE | p-value | Estimate | SE | p-value | Estimate | SE | p-value |
| Activity | 0.0011 | 0.0002 | 0.0047 | 0.0010 | 0.0002 | 0.0065 | 0.0011 | 0.0004 | 0.0240 | 0.0013 | 0.0004 | 0.0193 |
| Treatment | 0.3094 | 0.2393 | 0.2370 | -0.4101 | 0.1710 | 0.0476 | -0.2900 | 0.1702 | 0.1321 | -0.0570 | 0.1663 | 0.7416 |

The ANCOVA, a general linear model, was calculated in R studio for Pre-Infection, Night 4, Night 5, and Night 6. The regression included the predictor, independent, variables Activity and Treatment (control or infected) and the response, dependent, variable VO­_2_. Estimate indicated the mean change in response variable for a change in the predictor variable. SE = Standard Error.
